# Online Parenting Intervention for Children’s Eating and Mealtime Behaviors: Protocol of a Randomized Controlled Trial

**DOI:** 10.3390/healthcare10050924

**Published:** 2022-05-17

**Authors:** Vatsna Rathore, Amy E. Mitchell, Alina Morawska, Santosh Kumar Tadakamadla

**Affiliations:** 1 Discipline of Dentistry, Department of Rural Clinical Sciences, La Trobe Rural Health School, La Trobe University, Bendigo, VIC 3086, Australia; vatsna.rathore@griffithuni.edu.au; 2 School of Nursing and Midwifery, Griffith University, Brisbane, QLD 4222, Australia; amy.mitchell@griffith.edu.au; 3 Menzies Health Institute Queensland, Griffith University, Brisbane, QLD 4111, Australia; 4 Parenting and Family Support Centre, School of Psychology, The University of Queensland, Brisbane, QLD 4072, Australia; alina@psy.uq.edu.au; 5 Violet Vines Marshman Centre for Rural Health Research, La Trobe Rural Health School, La Trobe University, Bendigo, VIC 3550, Australia

**Keywords:** children, parents, healthy eating, snacking, intervention, study protocol

## Abstract

Introduction: Obesity and overweight are significant health problems among Australian children. Parents play a vital role in establishing healthy eating behaviors in their children. However, parents often experience difficulties in implementing effective parenting practices and lack confidence in their ability to help children adopt these behaviors. This trial will evaluate the efficacy of an online program, Healthy Habits Triple P, in improving children’s snacking and mealtime behaviors and related parenting practices. Methods and analysis: This is a single-blinded, randomized controlled trial for parents of young Australian children aged 2–6 years. Participants will be recruited through childcare centers, social media, online parent forums and existing networks. The participants in the intervention arm will receive access to a web-based parenting intervention in addition to nutrition-related information for parents published by the National Health and Medical Research Council of Australia; those in the control arm will receive nutrition-related information only. After the completion of the study, the parenting intervention will be offered to the control arm. The primary outcome will be improvement in children’s eating habits. The secondary outcomes include parents’ self-efficacy, confidence, children’s mealtime behaviors and mealtime parenting strategies. Both primary and secondary outcomes will be evaluated through online-administered, validated parent-reported questionnaires. We will also undertake a quantitative and qualitative evaluation of the practicality and acceptability of the intervention.

## 1. Introduction

The prevalence of *overweight* and *obesity* has risen from 4% in 1975 to 18% in 2016 among children and adolescents across the world [1]. Around 25% of Australian children and adolescents are currently overweight or obese, leading to worse health outcomes and poorer wellbeing [2]. Hayes et al. (2016) found that the direct healthcare costs of children with obesity aged 2–4 years was 1.62 times higher than healthy weight children [3]. The longer-term burden of disease from overweight and obesity is even greater, as it is a driver for multiple diseases including diabetes, musculoskeletal conditions, cardiovascular disease, kidney disease, asthma, dementia and various cancers in later life [4].

A complex interaction of biological, environmental and social factors influences the likelihood of a child gaining excess weight, such as differences in appetite and metabolism, accessibility of healthy food options and avenues for safe outdoor play [5,6]. Although the causes are complex, intake of energy-dense, nutrient-poor foods is a key contributor to obesity in Australian school children [7]. National guidelines recommend that children regularly consume a variety of fruits and vegetables, limit intake of treat and fast foods, consume water as the main drink and eat meals together with their family at the table [8,9]. Specific guidelines recommend that children consume a minimum of 1–2 servings of fruit and 2–3 servings of vegetables per day; however only 6% of children aged 5–14 years met these recommendations in 2017–2018 and almost half (45%) of the children aged 2–17 years regularly consumed sugar-sweetened food and/or diet drinks [10]. Among 2–3 year-old Australian children, discretionary food items (foods high in energy with saturated fat or added sugars or added salt) accounted for 29% and 32% of total energy intake/day for boys and girls, respectively [11]. Thirty-one percent of children are also likely to consume sugar-sweetened drinks on one to three days a week [11].

Parenting, parental role modelling and the home environment are important modifiable determinants of healthy eating in children [12,13]. Evidence from an observational study also demonstrated a cumulative model of association between family mealtime behaviors and child obesity status [14]. In this study, cumulative risk factors, such as family mealtime routines pertaining to regularity, roles and flexibility; and mealtime interactions pertaining to positive communication accounted for 12% of the variance in children’s body mass index [14]. The current study is framed around the importance of positive parenting practices that are proximal to establishing positive mealtime behaviors and communication.

The early years of life are also when children develop values, beliefs and attitudes, and adopt practices related to food intake [15]. Importantly, healthy practices adopted in childhood are more likely to continue into adolescence and adulthood [16].

A recent national survey conducted by the Royal Children’s Hospital Melbourne identified unhealthy diet as a top child health concern among Australian parents [17], yet many Australian children do not adhere to the recommended behavioural practices [18], as outlined in the national guidelines [8,9]. Within the family environment, both general and diet-specific parenting practices are related to children’s eating and mealtime behaviours [19]. General parenting styles are higher-order constructs, whereas parenting practices are more proximal in determining child behaviour [20]. For example, an authoritative parenting style is linked to a healthier weight and dietary outcomes in children, as compared to authoritarian, permissive or neglectful parenting styles [21]. Specific parenting practices, such as the types of food that parents make available to their children and parental role modelling, will tend to have a more direct effect on children’s eating behaviours [22].

A recent systematic review by Gomes et al. (2021) found that, despite a growing body of research examining the effects of parent-focused web-based interventions to promote children’s healthy diet, there is a paucity of studies examining the effects on parental feeding practices [23]. There is emerging evidence of web-based interventions improving parents’ knowledge, behaviour and attitudes in the context of child obesity and overweight [24,25]. Although most of the web-based interventions were theory-driven and included standard behaviour change techniques (e.g., instruction and demonstration of parenting behaviours) [26,27], few included strategies, such as the identification of oneself as a role model [28], promotion of children’s self-regulation or the management of parents’ emotional responses to children’s eating challenges, all of which are important to the development of healthy eating behaviours in childhood [29].

As a public health priority, government agencies recommend using primary and secondary prevention strategies to minimize the burden of diseases and associated risk factors [30]; however, despite these recommendations, a majority of parenting interventions for childhood obesity and overweight are treatment-based rather than prevention-oriented [25]. The Triple P (Positive Parenting Program) is a multi-level, prevention-oriented program, based on social learning principles and grounded in a self-regulatory framework, that takes a minimal sufficiency approach to support the development of parent and child self-regulation [31]. It aims to enhance parents’ knowledge, skills and confidence; promotes positive and nurturing environments; teaches strategies to support healthy child development and manage children’s problem behaviours; and supports parents with self-care and managing their own stress [32]. All of these factors have been identified as important to the development of healthy eating and mealtime behaviours in childhood [23]. The aim of this trial is to examine the efficacy of a prevention-based online intervention (Healthy Habits Triple P) designed to support parents to develop healthy eating and mealtime behaviours with their young children (2–6 years) in Australia.

## 2. Methods and Analysis

This protocol is reported in accordance with the guidelines presented in the SPIRIT 2013 statement for protocols of clinical trials [33].

### 2.1. Design

The proposed study is a randomized controlled trial to assess the efficacy of a web-based parenting support program for promoting healthy eating and mealtime behaviours. There will be two data collection time-points: baseline and post-intervention (6 weeks after the intervention). All participants in the intervention group will receive the web-based Healthy Habits Triple P intervention. The control group, on the other hand, will only have access to the online intervention after completing the six-week follow-up assessment. All parents will receive a brochure on child nutrition, produced by the Australian government (National Health and Medical Research Council (2021)) [34].

### 2.2. Participants and Setting

Any parent/caregiver interested in taking part in the program, living in Australia, and with at least one child in the 2–6 years age group is eligible to participate. Parents/caregivers will be included if they are able to read and understand English and have access to an internet-enabled device. Only one parent and child per family will be eligible to participate. For families with multiple children in the target age range, parents will be asked to nominate the child whose eating and mealtime habits they are most concerned about; otherwise, they will be asked to select their youngest child. Exclusion criteria are: (1) a child with a disability; (2) a child with behavioural difficulties requiring parents to seek professional help; and (3) parents currently receiving psychological support or counselling.

### 2.3. Sample Size

In this trial, following the recommendations of Whitehead et al. (2016), a sample size of 15 families per arm will be required with 90% power and two-sided 5% significance [35] and medium effect size (Cohen’s d = 0.5) based on meta-analysis of the effects of the Triple P on parenting and child outcomes [36]. Allowing for an attrition rate of 60%, 25 participants will be recruited to each arm. We have allowed for an attrition rate of 60%, which is greater than the traditional 20% attrition rate commonly used in trials of public health interventions. Participant anonymity in this study may contribute to reduced rates of assessment completion post-intervention [37]. An increase in sample size by 60% will ensure that sufficient sample size is retained.

### 2.4. Recruitment

Participants will be recruited using several approaches including (a) distribution of recruitment material through early childhood education and care services and playgroups using existing networks; and (b) social marketing and targeted advertisements through online social networks, such as Facebook and online parenting forums. A study flyer, including brief information about the study and a link to the study website, will be used to distribute study information to parents through the above networks. The website provides comprehensive information about the study, including eligibility criteria, and includes a link to the baseline online survey (developed using Lime Survey). Consenting parents will need to complete the online consent form before they can proceed to the survey questions. Recruitment of participants will continue until a sample of at least 50 participants is obtained. As a thank-you for their time and participation, all participants will receive a $20 retail voucher after completing their 6-week post-intervention questionnaire.

### 2.5. Randomisation, Allocation and Blinding

After the baseline survey is completed, participants will be randomly allocated to the intervention or the control groups in a 1:1 ratio using computer-generated block randomization schedule by a researcher not involved in data collection or recruitment, to facilitate equal group allocation. Because of the type of intervention used in this trial, participants cannot be blinded to group allocation.

### 2.6. Intervention

Triple P is a multilevel parenting and family support strategy developed by researchers at the University of Queensland, Australia. Self-regulation is the central construct of Triple P. The system takes a social learning approach to support healthy child development as well as to prevent and manage behavioural, social and emotional problems in children [31]. The intervention content focuses on reciprocal and bidirectional patterns of parent-child interaction and emphasizes positive and responsive parenting practices [38]. Parents are prompted to set up developmentally appropriate goals, have realistic expectations, create a safe, engaging and positive learning environment for children and use assertive discipline. The program is embedded within a self-regulatory framework which comprises a number of factors including self-sufficiency, self-efficacy and self-management, and is derived from social cognitive theory [39]. Further, parents’ capacity to monitor their own behaviour and that of their child is fundamental to achieving positive intervention outcomes [31].

Healthy Habits Triple P, tested in this study, is an online, self-directed intervention to help parents develop effective parenting practices and increase their confidence in managing children’s eating and mealtime behaviours. The intervention consists of two modules. Module 1 consists of eight sections covering evidence-based positive parenting strategies in the context of developing healthy habits with young children, including strategies for encouraging healthy habits (e.g., having realistic expectations and use of specific instructions, praise, reward charts and routines), dealing with resistance, planning for high-risk situations, building children’s independence, parental coping and using evidence to make informed decisions about children’s health. Module 2 has four sections focusing on the application of positive parenting strategies to eating and mealtime behaviours (e.g., encouraging healthy food choices, increasing food variety, encouraging self-regulation and dealing with problem eating and mealtime behaviours). This section focuses on formulating individual parent- or child-focused goals and applying relevant parenting strategies to reach these goals. The intervention takes approximately 4–5 hours to complete, with parents encouraged to complete the modules over a two-week period. Module content is tailored towards key concerns and difficulties commonly experienced by the parents of young children. The modules include psychoeducation alongside the text, audio and videos guiding parents in the use of specific parenting techniques. The intervention has been developed to engage parents by including narratives and examples to help parents relate the material to their own lives.

### 2.7. Data Collection

All data will be collected using validated parent-reported questionnaires that are to be completed online. Parents will complete the baseline survey soon after recruitment into the study and the post-intervention survey 6 weeks after randomization. Both the baseline and post-intervention surveys will be hosted on the Lime Survey platform. In order to minimize attrition, email reminders will be sent to parents to complete the modules and the post-intervention survey. The prompts will be sent out on a weekly basis after the participants are randomized. Additional reminders will be sent to families where post-intervention assessments have not been completed by the due date. The control group will complete the same set of assessments at the same time points.

Family socio-demographic information will be collected at baseline using the Family Background Questionnaire [40], including the child’s age, sex, postcode, the relationship of the respondent to the child, marital status, family structure, ethnicity, number of people living in the house, level of education, work status and country of birth of the respondent and their partner and the family’s ability to meet essential expenses.

### 2.8. Primary Outcomes

The efficacy of the intervention will be measured through parent-reported changes in children’s diets using the validated Children’s Dietary Questionnaire (CDQ) [41]. The CDQ is a 28-item questionnaire that assesses children’s dietary intake patterns over the past week (or 24 h) for foods and beverages which are recommended or discouraged for health reasons. The tool was developed based on the Australian healthy eating guidelines and has demonstrated acceptable reliability and validity for assessing dietary patterns among preschool children at a population level [42]. It has been recommended for use in evaluating the efficacy of interventions to improve children’s eating habits [42]. All subscales of CDQ had Cronbach’s alpha (α) values of ≥0.6 [42].

### 2.9. Secondary Outcomes

Parents’ confidence in dealing with children’s demands for sugar-rich foods will be assessed using the Comprehensive Snack Parenting Questionnaire (CSPQ). The CSPQ is a parent-reported questionnaire comprising 21 items with a five-point Likert response scale ranging from ‘strongly disagree’ to ‘strongly agree’ [43]. In an Australian study with 134 participants, the Cronbach’s α value of the total scale was 0.752 [44]. In addition, parents’ self-efficacy for promoting healthy eating and limiting non-core foods will be evaluated using nine items from a previous Australian study [45]. Self-efficacy for promoting healthy eating is measured by five items assessing parents’ confidence in their ability to get their child to eat healthily (e.g., “How confident are you that you could get your baby/child to eat enough fruit over the next year?”) and a further four items assess parents’ confidence in refusing their child’s requests for unhealthy/non-core foods (e.g., “How confident are you that you could say no to your baby/child’s demands/fussing for sweet snacks, confectionary, lollies and/or ice cream?”). The responses are rated on a five-point Likert scale ranging from ‘not confident at all−1′ to ‘very confident−5′, with higher scores indicating higher levels of self-efficacy. The self-efficacy scale was found to be reliable (α = 0.86) [43].

Lastly, parents will complete the child feeding behaviour and mealtime parenting strategies scales of the Parent and Toddler Feeding Assessment (PATFA), a parent-report questionnaire used to assess child behaviour, parenting confidence and parenting strategies relevant to child feeding and mealtimes [46]. Parents will be asked to rate the frequency of 16 common child feeding problems (e.g., spitting food out) on a five-point scale from 1 (never) to 5 (almost always) and whether each behaviour was problematic (yes/no), followed by their confidence in managing each behaviour on a 10-point scale (with higher scores indicating greater confidence). Parents will also rate how frequently they used each of 29 parenting strategies at mealtimes (e.g., eating with the child) on a five-point scale from 1 (never) to 5 (almost always). The subscales of PATFA have good test–retest reliability (*r*  =  0.68–0.89) and excellent internal consistency (α  =  0.65–0.97).

### 2.10. Practicality and Acceptability

Practicality will be assessed by determining the rate of completion (number of participants accessing and completing all the modules of the intervention) and data collected remotely from the online intervention platform. Time required to recruit the estimated sample will also be estimated [47].

Acceptability of the intervention will be assessed both through open-ended questions and qualitative interviews. The three open-ended questions, such as “Do you have any other comments about the program or any suggestions about how we could improve the program for families in the future”, will help evaluate the perceived usefulness of online modules. Personal interviews will also assist in obtaining parents’ perception of the usefulness of the content, barriers and facilitators of using the intervention. We will evaluate parents’ satisfaction with the intervention using the Client Satisfaction Survey [48]. The survey consists of 10 items which are rated using a seven-point Likert response scale (e.g., “Has the program helped you to deal more effectively with your child’s eating or mealtime behaviour?”)

### 2.11. Quantitative Data Analysis

Quantitative data from this pilot study will be descriptive, with outcomes being interval estimates of variables relating to children’s dietary habits, parenting and child behaviours and parenting efficacy. Data will be de-identified, and SPSS (Version 27.0. Armonk, NY, USA: IBM Corp) will be used to conduct data analysis. Data will be examined for outliers and missing values. All data will be analyzed based on the intention to treat (ITT) principle. To account for repeated measurements, two-way repeated-measures ANOVAs will be used to evaluate changes in diet and other study outcomes between the intervention and control groups.

### 2.12. Qualitative Data Analysis

For qualitative data, parent interviews will be audio-recorded and transcribed verbatim, and transcripts will be analyzed. A two-stage process will be followed to analyze interview transcripts. The first interview transcript will be pilot coded independently by two researchers to agree on a coding strategy (i.e., to ensure both researchers are coding consistently and to discuss and resolve any difficulties). Initial findings of the pilot transcript will be discussed before the researchers independently code the remaining transcripts. This will involve reading and re-reading transcripts, coding the content into themes and sub-themes and mapping these, with supporting direct quotes. The sample size for interviews will be determined based on data saturation.

## 3. Discussion

This study will examine the efficacy of Healthy Habits Triple P, an online intervention developed to improve children’s snacking and mealtime behaviours and related parenting practices. Healthy Habits Triple P has been developed in response to research identifying parents as important for behaviour maintenance and behaviour change in relation to their children’s snacking and mealtime behaviours [49]. Interventions that aim to alter the child’s eating behaviours often focus on parents as a proximal influence [50]. Prior interventions have varied in content, such as programs that seek to increase parental awareness of their child’s obesity risk [51], encouraging regular family routines around mealtimes, sleep, and/or media use [52], and family-based obesity prevention strategies that seek to engage the whole family unit [53] or intervention approaches that focus more directly on parents’ own weight management and nutrition [54].

Online administered, preventive public health interventions addressing lifestyle-associated disease risk factors could be cost-effective and could easily be scaled up. A recent systematic review by Gomes et al. (2020) evaluated the effectiveness of web-based interventions in changing parental feeding practices [23]. The review demonstrates that several intervention elements that are central to social cognitive theory, such as ‘*goals and planning*’ and ‘*feedback and monitoring*’, have been infrequently incorporated into interventions to date [26]. There is also evidence that healthy eating and physical activity interventions that combine self-monitoring with at least one other self-regulatory strategy tend to be more effective than programs that do not include these elements [55]. Healthy Habits Triple P builds upon previous research in this area by incorporating important drivers of behaviour change, including ‘goal setting’, ‘monitoring of parent and child behaviour’ and parent ‘self-reflection’. It aims to develop parents’ capacity for self-regulation as a central skill, resulting in parents becoming independent problem solvers and being able to implement parenting strategies effectively.

The systematic review by Gomes et al. (2020) also found that most parent-focused web-based interventions did not improve diet-related parenting practices, except for food availability [23]. These negligible effects could be attributed to the inclusion of active control groups which, in many studies, received content that was overly similar to that provided to the intervention groups. In our trial, a waitlist control group will be used to maximize treatment separation and help prevent the problem of diluted effect, reported in previous studies.

It is important for parent-focused interventions: to acknowledge that each child has different choices and behaviours related to eating; to address parents’ own beliefs, attitudes and goals related to healthy eating; and to promote strategies to address other family factors, such as involving other caregivers [56]. Our intervention tailors to individual circumstances by helping parents to have realistic expectations of their child and themselves, given their child’s age and stage of development; to develop child-focused goals that are based on their own individual needs and priorities; and to develop and maintain effective daily routines by planning ahead and involving other family members.

One potential limitation of the study is the reliance on parent-reported measures for outcome assessment. However, at minimum, the results from this study will provide a good indication of whether an intervention such as this is practical to deliver and acceptable to parents. Future studies should explore the concordance of parent-reported parenting and child behaviour outcomes with observed measures of parent and child behaviour. This study provides the opportunity to estimate important study-specific parameters required to inform the design of future definitive controlled trials [57] which, if successful, will pave the way toward implementation as a preventive public health intervention within existing healthcare settings.

## Data Availability

Not applicable.
